# Supplementary figures and images for: A scalable method to improve gray matter segmentation at ultra high field MRI
Source: PLoS One. 2018 Jun 6;13(6):e0198335. doi: 10.1371/journal.pone.0198335 (PMC5991408; doi:10.1371/journal.pone.0198335)

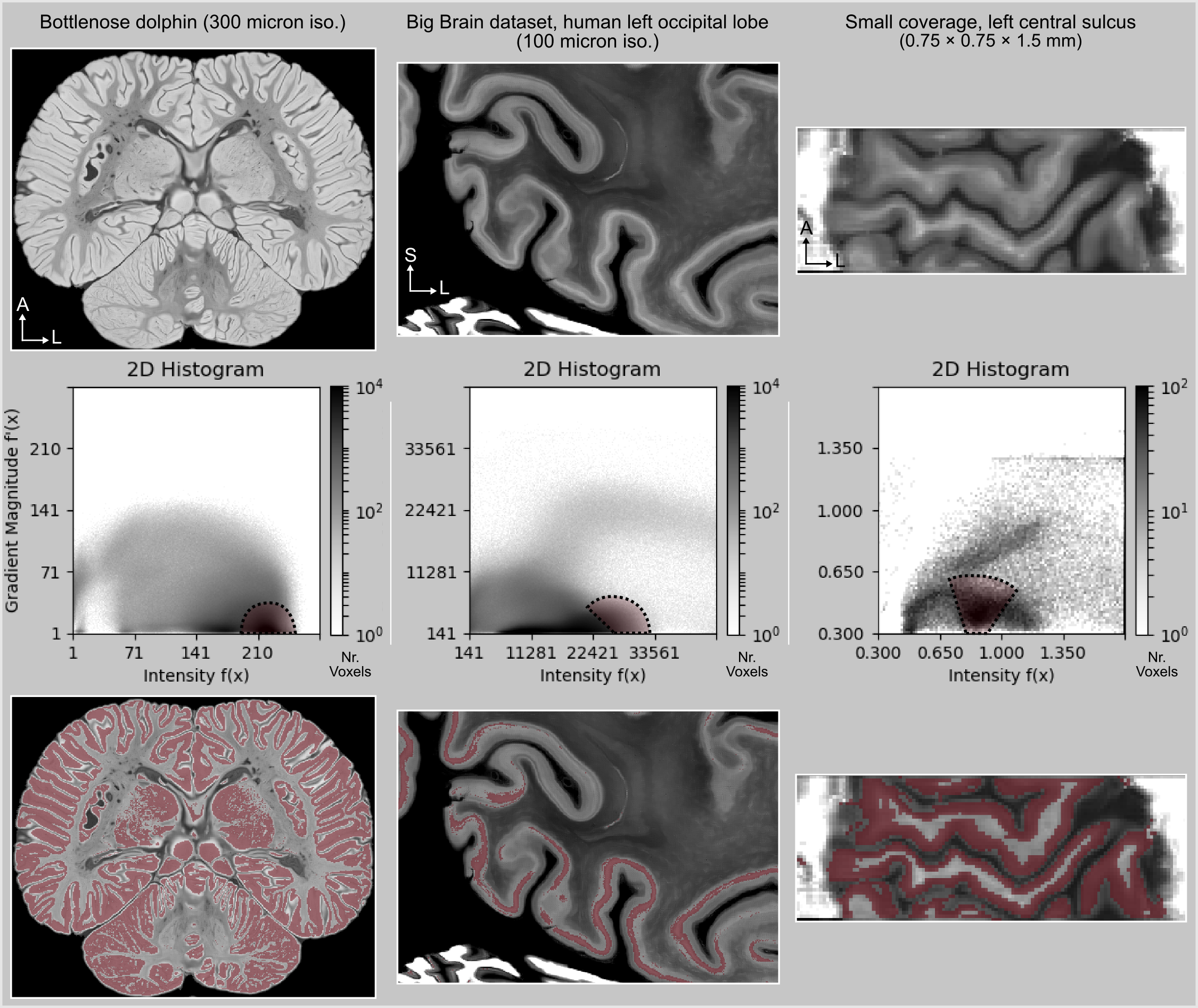

Supplement: S6 Fig — Shown are several examples of the variety of existing volumetric datasets for which our methods appear to be useful. Every column represents different images: the brain of a bottle-nose dolphin [92] (left), the occipital lobe of a human brain with 100 micron resolution [93] (middle) and a human motor cortex acquired with small partial coverage (T1w EPI) with anisotropic resolution [94] (right). For every image we show a slice (top row), selected voxels in the 2D histogram (middle row) and selected voxels overlaid on the slice (bottom row). These images do not contain large intensity inhomogeneities. Therefore, no bias-field correction was performed. Mild non-linear anisotropic diffusion-based smoothing was applied to enhance CNR. (TIFF) [file pone.0198335.s006.tiff]

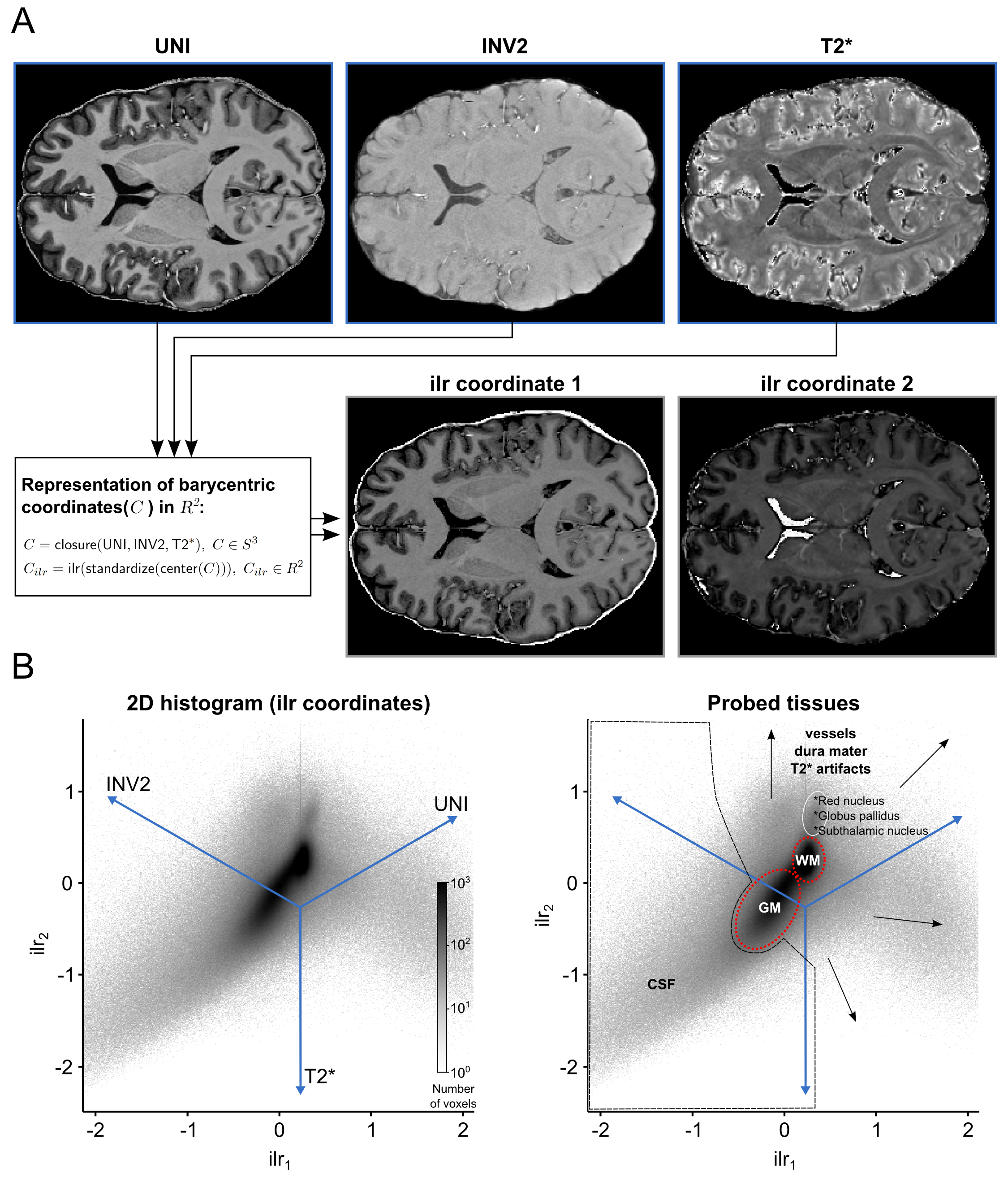

Supplement: S7 Fig — (A) Each voxel is considered as a three part composition. The barycentric coordinates of each composition which reside in 3D simplex space are represented in 2D real space after using a isometric log-ratio (ilr) transformation. (B) The ilr coordinates are used to create 2D histograms representing all voxels in the images. The blue lines are the embedded 3D real space primary axes (note that the input image units were initially normalized to have similar dynamic ranges to account for the large scale difference between T2* and MP2RAGE images). In this case, the ilr coordinates are not easily interpretable by themselves but they are useful to visualize the barycentric coordinates which are interpretable via the embedded real space axes. Darker regions in the histogram indicate that many voxels are characterized by this particular scale invariant combination of the image contrasts. In this representation, brain tissue (WM and GM, red dashed lines) becomes separable from non-brain tissue (black dashed lines and arrows). If desired, subcortical structures like the red nucleus, the globus pallidus and the subthalamic nucleus can additionally be identified (white circle). (TIFF) [file pone.0198335.s007.tiff]

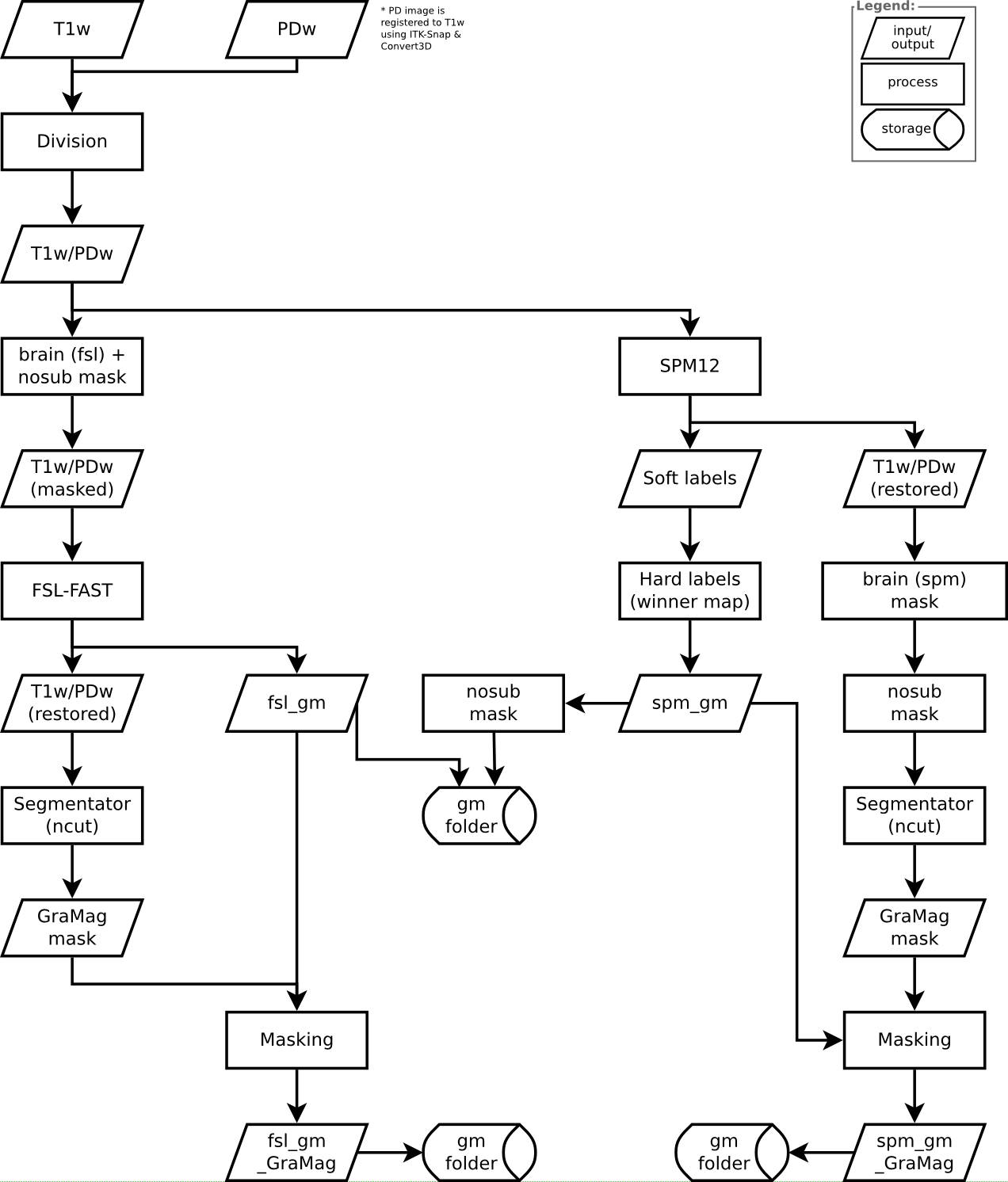

Supplement: S8 Fig — This diagram provides a detailed overview of all the inputs, processing steps and outputs for MPRAGE GraMag pipeline. Rectangular shapes represent processing steps, rhombic shapes represent input or outputs and cylindrical shapes represent input or output locations. (TIFF) [file pone.0198335.s008.tiff]

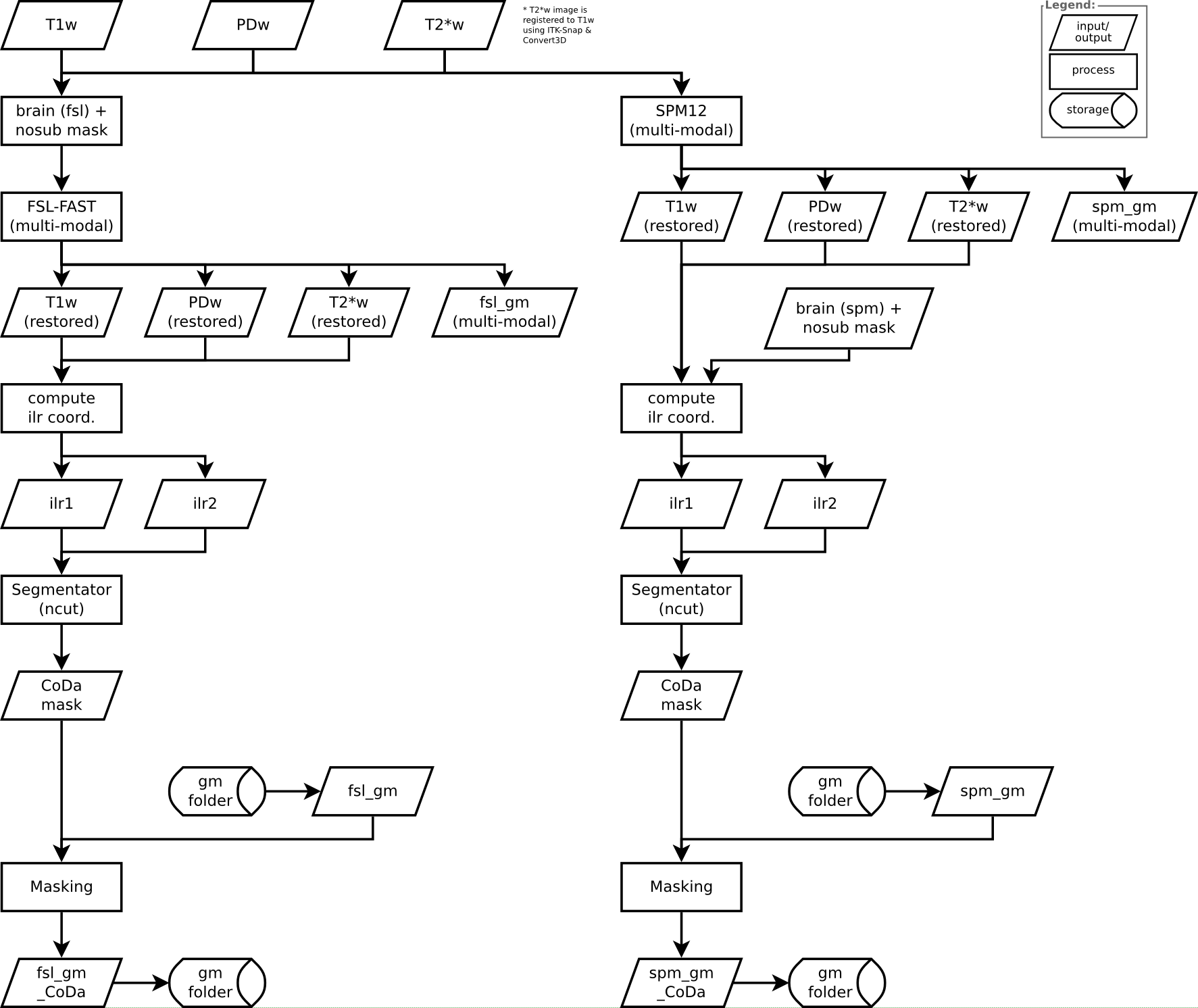

Supplement: S9 Fig — This diagram provides a detailed overview of all the inputs, processing steps and outputs for MPRAGE CoDa pipeline. Rectangular shapes represent processing steps, rhombic shapes represent input or outputs and cylindrical shapes represent input or output locations. (TIFF) [file pone.0198335.s009.tiff]

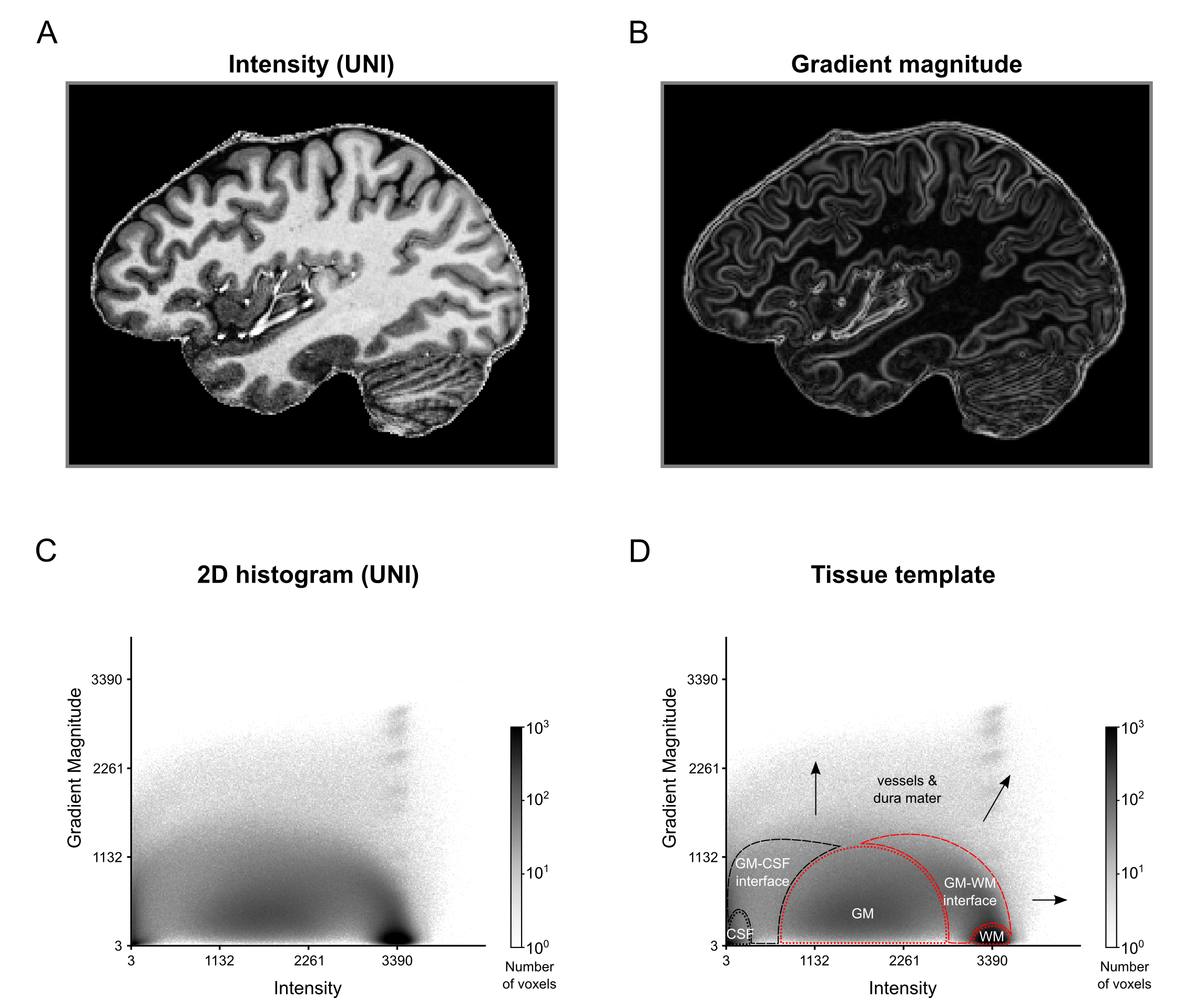

Supplement: S12 Fig — The intensity (A) and gradient magnitude (B) values of a T1w-divided-by-PDw MRI image (MP2RAGE, 0.7 mm isotropic resolution) are represented in a 2D histogram (C). Darker regions in the histogram indicate that many voxels are characterized by this particular combination of image intensity and gradient magnitude. The 2D histogram displays a characteristic pattern with tissue types occupying particular areas of the histogram (D). Voxels containing CSF, dura mater or blood vessels (black dashed lines and arrows) cover different regions of the histogram than voxels containing WM and GM (red dashed lines). As a result, brain tissue becomes separable from non-brain tissue. (TIFF) [file pone.0198335.s012.tiff]
