# Supplementary material for: A scalable method to improve gray matter segmentation at ultra high field MRI
Source: PLoS One. 2018 Jun 6;13(6):e0198335. doi: 10.1371/journal.pone.0198335 (PMC5991408; doi:10.1371/journal.pone.0198335)
Supplement: S1 Appendix — (PDF) [file pone.0198335.s013.pdf]

Time benefit estimation. To estimate the time saved by substituting manual correction with Segmentator, we did the following calculation: Assuming that the objective of manually correcting mislabeled GM voxels is to reduce the number of false positives and to increase the number of true positives, we computed the number of false and true positives both before and after Segmentator intervention. We computed the difference in true positives and false positives before and after the application of segmentator and subtracted the true positive difference from the false positive difference. The resulting number is assumed to indicate the number of voxels which would have to be subtracted in the process of manually correcting all the voxels without using Segmentator. For the MRI data presented here, this number amounted to around 200000 voxels to be corrected (out of a total number of 1100000 voxels in the cortical ribbon). On average 35000 out of those 200000 voxels could be corrected using Segmentator. Assuming that a trained operator can manually correct one voxel per second, on average, this amounts to 7.5 hours of manual work that can be substituted with 10 minutes of Segmentator usage for the whole brain GM ribbon segmentation at 0.7 mm isotropic resolution. The time that can potentially be saved by using Segmentator will scale with the total number of GM voxels - it will be higher for high resolution acquisition (more voxels) and lower at low resolution (less voxels).
