# Supplementary material for: A scalable method to improve gray matter segmentation at ultra high field MRI
Source: PLoS One. 2018 Jun 6;13(6):e0198335. doi: 10.1371/journal.pone.0198335 (PMC5991408; doi:10.1371/journal.pone.0198335)
Supplement: S1 Table — (PDF) [file pone.0198335.s015.pdf]

S1 Table. Segmentation performance MPRAGE data without artifact masking. The table shows the DICE (larger is better) and AVHD (less is better) for the initial SPM 12 and FSL FAST GM segmentations as well as after additional polishing, using either the gradient magnitude or the compositional data method.

		SPM		FAST	
		DICE	AVHD	DICE	AVHD
S02					
	Init	0.8576	0.7413	0.8896	0.5375
	Init + GraMag	0.8594	0.5865	0.8902	0.4980
	Init + CoDa	0.8525	0.6061	0.8421	0.5557
S03					
	Init	0.8644	0.8753	0.8694	0.5739
	Init + GraMag	0.8376	0.6139	0.8781	0.5612
	Init + CoDa	0.8483	0.6299	0.8386	0.5687
S05					
	Init	0.8569	0.6379	0.8782	0.5230
	Init + GraMag	0.8688	0.5647	0.8909	0.5233
	Init + CoDa	0.8596	0.5591	0.8692	0.5150
S06					
	Init	0.8601	0.7789	0.8635	0.6477
	Init + GraMag	0.8435	0.5797	0.8616	0.5909
	Init + CoDa	0.8442	0.6287	0.8580	0.5530
S07					
	Init	0.8897	0.6403	0.8490	0.7228
	Init + GraMag	0.8893	0.5574	0.8460	0.6862
	Init + CoDa	0.8508	0.5974	0.7935	0.7305

DICE, DICE Coefficient; AVHD, Average Hausdorff Distance; GraMag, gradient magnitude method; CoDa, compositional data method.
