# Supplementary material for: A scalable method to improve gray matter segmentation at ultra high field MRI
Source: PLoS One. 2018 Jun 6;13(6):e0198335. doi: 10.1371/journal.pone.0198335 (PMC5991408; doi:10.1371/journal.pone.0198335)
Supplement: S2 Table — (PDF) [file pone.0198335.s016.pdf]

S2 Table. Segmentation performance MP2RAGE data without artifact masking. The table shows the DICE (larger is better) and AVHD (less is better) for the initial CBS tools and FSL FAST GM segmentations as well as after additional masking, using either the gradient magnitude or the compositional data method.

		CBS		FAST	
		DICE	AVHD	DICE	AVHD
S001					
	Init	0.8688	0.4081	0.8157	1.0427
	Init + GraMag	0.9032	0.4538	0.8095	0.8534
	Init + CoDa	0.8914	0.3925	0.8520	0.6290
S013					
	Init	0.8451	0.6146	0.7539	1.0551
	Init + GraMag	0.8501	0.5377	0.7363	0.9299
	Init + CoDa	0.8398	0.5270	0.7787	0.7613
S014					
	Init	0.8389	0.6730	0.8089	0.9837
	Init + GraMag	0.8410	0.5532	0.7868	0.7996
	Init + CoDa	0.8485	0.5424	0.8213	0.7258
S019					
	Init	0.8920	0.4798	0.8356	0.9672
	Init + GraMag	0.8794	0.3639	0.8081	0.7359
	Init + CoDa	0.8772	0.4365	0.8499	0.6204

DICE, DICE Coefficient; AVHD, Average Hausdorff Distance; GraMag, gradient magnitude method; CoDa, compositional data method.
